# Dithranoltherapie bei Psoriasis: eine Pilotstudie zur in vivo konfokalen Laserscanmikroskopie

**DOI:** 10.1111/ddg.15825_g

**Published:** 2025-10-23

**Authors:** Julia K. Winkler, Ferdinand Toberer, Astrid Schirra‐Hoffmann, Lena Vogelgsang, Alexander Enk, Holger A. Haenssle

**Affiliations:** ^1^ Hautklinik Universitätsklinikum Heidelberg, Heidelberg

**Keywords:** Dithranol, konfokale Laserscanmikroskopie, Psoriasis, Dithranol, psoriasis, reflectance confocal microscopy

## Abstract

**Hintergrund:**

Histomorphologische Daten zum Ansprechen von Psoriasisläsionen auf topisches Dithranol sind nur begrenzt verfügbar. Befunde der in vivo konfokalen Laserscanmikroskopie (KLM) in Psoriasisläsionen sind hoch korreliert mit denen der Histopathologie und erlauben die nichtinvasive Beurteilung von Behandlungseffekten auf zellulärer Ebene.

**Patienten und Methodik:**

Prospektive, monozentrische Pilotstudie an einer Universitätshautklinik zwischen 01.01. und 30.08.2016. Psoriasisläsionen von 20 Patienten wurden mit Dithranol behandelt und vor Beginn sowie an den Tagen 4 und 8 der Behandlung mittels KLM untersucht.

**Ergebnisse:**

KLM‐Untersuchungen der Psoriasisläsionen unter Dithranoltherapie zeigten epidermale histomorphologische Veränderungen mit deutlicher medianer Reduktion der Hyperkeratose um 45,0% (*p* < 0,001), der Akanthose um 38,2% (*p* < 0,001) und der Epidermisdicke um 66,5% (*p* < 0,001) vom Ausgangswert bis zum Tag 8. Weiterhin ergab die semiquantitative Analyse der Parakeratose eine signifikante Reduktion bis Tag 8 (*p* < 0,001). Korrespondierend zeigte die KLM dermale histomorphologische Veränderungen mit Abnahme des Durchmessers der dermalen Papillen um 32,1% (*p* < 0001), Abnahme des Durchmessers der Papillengefäße um 16,9% (*p*  =  0,002) und deutlicher semiquantitativer Reduktion des Entzündungsinfiltrats (*p* < 0,001).

**Schlussfolgerungen:**

Die Ergebnisse unserer Pilotstudie deuten darauf hin, dass die topische Dithranoltherapie von Psoriasisläsionen zu starker und signifikanter Reduktion der pathologischen epidermalen und dermalen RCM‐Merkmale führen kann.

## EINLEITUNG

Psoriasis vulgaris ist eine multifaktorielle chronisch entzündliche Hauterkrankung mit einer weltweiten Inzidenz von 2%.^1^ Psoriasisläsionen sind gekennzeichnet durch scharf begrenzte erythrosquamöse Plaques an den Streckseiten der Extremitäten, am Kopf, Abdomen und sakral.^1^ In der klinischen Routine wird die Diagnose Psoriasis nach visueller Inspektion und Berücksichtigung der individuellen und Familienanamnese gestellt. In klinischen Studien oder schwer zu diagnostizierenden Fällen kann eine histopathologische Untersuchung notwendig sein. Die histomorphologischen Charakteristika der Psoriasis vulgaris umfassen konfluierende Hyperparakeratose, regelhafte Akanthose mit elongierten Reteleisten, Hypogranulose (fehlendes oder reduziertes Stratum granulosum), Ausdünnung der suprapapillären Ebene, Munro‐Mikroabszesse (Ansammlungen von Neutrophilen im Stratum spinosum), dilatierte Kapillaren in den dermalen Papillen sowie elongierte dermale Papillen mit dermalem Entzündungsinfiltrat. Abhängig von der Ausprägung der Hautveränderungen, der Compliance des Patienten, dem Vorliegen einer Psoriasisarthritis und Komorbidität können behandelnde Ärzte zwischen topischen und systemischen Therapieoptionen wählen. Während die Auswahl an systemischen Therapien groß ist und weiter zunimmt, zählen topische Steroide, Vitamin‐D_3_‐Analoga – als Monopräparate oder in Fixkombination mit Kortikosteroiden – sowie Dithranol zu den am häufigsten verwendeten topischen Therapien.^2^, ^3^

Dithranol, das vor über 90 Jahren eingeführt wurde, induziert rasche und ausgeprägte lokale Reaktionen, die mit einem deutlichen Erythem an den Applikationsstellen einhergehen.^4^, ^5^ Diese dosisabhängige Hautreaktion ist Voraussetzung für den therapeutischen Effekt auf sowohl den hyperproliferativen als auch den inflammatorischen Aspekt der Psoriasis. Gleichzeitig stellt das lokale Erythem mit brennenden Missempfindungen eine der limitierenden Nebenwirkungen dar.^6^ Trotz der exzellenten therapeutischen Wirksamkeit von topischem Dithranol gibt es nur begrenzte Daten zum mikrometrischen und morphologischen Einfluss auf die psoriatische Haut.^7^ Wir haben hierzu eine Pilotstudie zur Untersuchung der Veränderungen in psoriatischer Haut nach wiederholter Behandlung mi Dithranol durchgeführt. Zur Analyse identischer Lokalisationen innerhalb von Psoriasisläsionen setzten wir die nichtinvasive in vivo konfokale Laserscanmikroskopie (KLM) ein, die eine *en‐face*‐Bildgebung in zellulärer Auflösung entlang der horizontalen Schnittebene ermöglicht. Ziel dieser Pilotstudie war, die dynamischen Veränderungen in Psoriasis vulgaris, die wiederholt topisch mit Dithranol behandelt wurde, mittels KLM zu untersuchen.

## PATIENTEN UND METHODEN

### Patienten and Studiendesign

Dies ist eine monozentrische, prospektive Pilotstudie. Sie wurde von der lokalen Ethikkommission genehmigt und in Übereinstimmung mit der Deklaration von Helsinki durchgeführt. Alle Patienten gaben vor Studieneinschluss ihr schriftliches Einverständnis. Eingeschlossen wurden Patienten mit Psoriasis vulgaris im Alter von mindestens 18 Jahren und mit gegebener Indikation zur stationären topischen Dithranoltherapie im Zeitraum vom 01.01. bis 30.08.2016. Für jeden Patienten wurde eine Zielläsion ausgewählt und mithilfe digitaler Dermatoskopie sowie konfokaler Laserscanmikroskopie (KLM; VivaScope 1500, Caliber Imaging & Diagnostics, Rochester, NY, USA) zu Studienbeginn sowie an Tag 4 und Tag 8 der Dithranoltherapie dokumentiert. Die Zielläsionen waren an für KLM leicht zugänglichen Körperstellen lokalisiert und zeigten typische klinische und dermatoskopische Charakteristika der Psoriasis. Zur Dokumentation wurden digitale dermatoskopische Aufnahmen vor jeder KLM‐Messung in gleicher Lokalisation durchgeführt. Vor Studienbeginn wurden alle Patienten mit 10,0% Salicylsäure in weißer Vaseline zur Schuppenlösung vorbehandelt. Die Dithranoltherapie wurde mit 1/16% in weißer Vaseline einmal täglich begonnen (Dithranol 0,0625 g, Salicylsäure 3,65 g, weiße Vaseline ad 100 g). Die Dithranolkonzentrationen wurden individuell gesteigert (auf jeweils 0,125%, 0,25% und 0,5%) mit dem Ziel, das durch Dithranol induzierte lokale Erythem aufrechtzuerhalten. Keiner der Patienten erhielt innerhalb eines Monats vor Studienbeginn eine andere antipsoriatische Therapie.

### KLM‐Bildgebung

Für die KLM‐Untersuchungen wurde das VivaScope 1500‐Gerät (Caliber Imaging & Diagnostics, Rochester, NY, USA) entsprechend einem standardisierten Protokoll eingesetzt. Die VivaBlock™‐Software wurde verwendet, um vertikal gestapelte Bilder entlang der z‐Achse aufzunehmen – beginnend von der oberen Epidermis im Zentrum der Psoriasisläsion bis zur dermoepidermalen Junktionszone (DEJ) beziehungsweise oberen Dermis. Die entsprechenden Aufnahmen wurden analysiert, jeweils erhoben zu Studienbeginn sowie an Tag 4 und Tag 8: *(1)* drei z‐Stapel von der Oberfläche des Stratum corneum bis in eine Tiefe von 180 µm in Schritten von 4,5 µm sowie *(2)* vier Mosaike mit einer Ausdehnung von mindestens 4  ×  4 mm auf Höhe des Stratum corneum, des Stratum granulosum/spinosum, der DEJ und der papillären Dermis. Alle Aufnahmen wurden verwendet, um systematisch das Vorhandensein, das Ausmaß und die exakten Maße (in µm) prädefinierter KLM‐Merkmale zu untersuchen (Tabelle 1).

**TABELLE 1:** Übersicht über definierte mikrometrische und morphologische Kriterien in Zielläsionen.

**Histopathologische Merkmale**	**Beschreibung der Kriterien in KLM**
Hyperkeratose in µm	Mittlerer Abstand aus 3 Messungen von der Epidermisoberfläche bis zum Honigwabenmuster entlang der Z‐Achse innerhalb der Zielläsion
Akanthose in µm	Mittlerer Abstand aus drei Messungen vom Honigwabenmuster bis zum Ende der Reteleisten entlang der Z‐Achse innerhalb der Zielläsion
Epidermisdicke in µm	Mittlerer Abstand aus drei Messungen von der Epidermisoberfläche bis zum Ende der Reteleisten entlang der Z‐Achse innerhalb der Zielläsion
Parakeratose (nicht, moderat oder ausgeprägt vorhanden)	Semiquantitative Untersuchung hellerer rundlicher Strukturen innerhalb des Stratum corneum, die hellen Reste von Keratinozytenkernen enthalten manchmal umgeben von einem dunkleren Halo Anzahl der Kerne (Mittel aus 5 Sichtfeldern, Viva‐Stack 500 × 500 µm) ‐ nicht (0 Zellen) ‐ moderat (1–10 Zellen) ‐ ausgeprägt vorhanden (> 10 Zellen)
Munro‐Microabszesse (nicht, moderat oder ausgeprägt vorhanden)	Semiquantitative Untersuchung gruppierter, hellerer, polymorpher Zellen am Übergang des Honigwabenmusters zum Stratum corneum Anzahl der Mikroabszesse (Mittel aus 5 Sichtfeldern) ‐ nicht (0) ‐ moderat (1–10) ‐ ausgeprägt vorhanden (> 10)
Länge der dermalen Papillen in µm	Mittlerer Abstand aus drei Messungen vom Pflastersteinmuster der suprapapillären Ebene bis zum Ende der Reteleisten entlang der Z‐Achse innerhalb der Zielläsion
Durchmesser der dermalen Papillen in µm	Mittlerer innerer Durchmesser von drei dermalen Papillen von 3 Messpunkten innerhalb der Zielläsion (insgesamt 9 Messungen)
Vergrößerte papilläre Gefäße (nicht, moderat oder ausgeprägt vorhanden)	Semiquantitative Erfassung prominenter runder oder linearer, dunkler kanalikulärer Strukturen mit dünnwandiger Umgrenzung innerhalb der dermalen Papillen auf Höhe der papillären Dermis. Zahl vergrößerter Gefäße > 80 µm (Mittel aus 5 Sichtfeldern) ‐ nicht (0) ‐ moderat (1–10) ‐ ausgeprägt vorhanden (> 10)
Durchmesser der papillären Gefäße in µm	Mittlerer innerer Durchmesser von 3 papillären Gefäßen von 3 Messpunkten innerhalb der Zielläsion (insgesamt 9 Messungen)
Entzündungsinfiltrat (nicht, moderat oder ausgeprägt vorhanden)	Semiquantitative Untersuchung von kleinen homogen hellen Zellen vereinbar mit Lymphozyten Anzahl der Lymphozyten (Mittel aus 5 Sichtfeldern) ‐ nicht (0) ‐ moderat (1–25) ‐ ausgeprägt vorhanden (> 25)

### Histopathologie

Bei zwei Patienten wurden repräsentative 4‐mm‐Stanzbiopsien an den Stellen der jeweiligen KLM‐Messungen zu Studienbeginn sowie an den Tagen 4 und Tag 8 durchgeführt (jeweils drei Biopsien pro Patient). Die Entnahmen erfolgten unmittelbar nach den KLM‐Messungen aus identischen Psoriasisplaques. Es wurden Hämatoxylin‐Eosin (HE)‐Färbungen angefertigt. Die Biopsien dienten der Korrelation der KLM‐Merkmale mit histopathologischen Befunden. Die histopathologischen Untersuchungen wurden exemplarisch nur bei zwei Patienten durchgeführt, um die Zahl invasiver Eingriffe zu begrenzen.

### Statistische Analysen

Die erhobenen Daten wurden mittels deskriptiver Statistik ausgewertet; entsprechende Grafiken veranschaulichen die Ergebnisse. Absolute Zahlen und Medianwerte wurden zur Darstellung der KLM‐Messungen verwendet. Zur Abbildung der Veränderungen über die Zeit wurden normalisierte Prozentangaben genutzt, wobei der Median zu Studienbeginn 100% entsprach. Zwanzig Patienten wurden als ausreichend erachtet, um alle deskriptiven Zielsetzungen dieser Pilotstudie zu erfüllen. Zur Analyse signifikanter dynamischer Veränderungen in den absoluten Messwerten über die Zeitpunkte Studienbeginn, Tag 4 und Tag 8 wurde der Friedman‐Test angewendet. Ergebnisse wurden bei p < 0,05 als statistisch signifikant gewertet. Bonferroni‐Korrekturen für multiples Testen wurden bei Bedarf berücksichtigt. Alle Analysen erfolgten mit SPSS, Version 29 (IBM, SPSS, Chicago, IL, USA).

## ERGEBNISSE

Es wurden 20 Patienten rekrutiert (12 Männer, 8 Frauen) mit einem medianen Alter von 53,5 Jahren (Bereich: 18 bis 75 Jahre). Zum Studienbeginn lag der mediane *Psoriasis Area and Severity Index* (PASI) der Teilnehmer bei 12,9 (Bereich: 10,1 bis 18,0). Die psoriatischen Zielläsionen für die KLM‐Messungen und Biopsien waren überwiegend an den Oberschenkeln lokalisiert (30%), gefolgt von Unterarmen und Unterschenkeln (jeweils 25%). Weitere anatomische Lokalisationen wurden seltener einbezogen, darunter Handrücken (10%), Abdomen (5%) und Rücken (5%) (Tabelle 2).

**TABELLE 2:** Patientencharakteristika.

	Median	Range
Medianes Alter	53,5	18–75
Medianer PASI	12,9	10,1–18,0
	***n***	***%***
Geschlecht		
Weiblich	8	40,0
Männlich	12	60,0
Läsionslokalisation		
Rumpf	2	10,0
Unterarm	5	25,0
Hand	2	10,0
Oberschenkel	6	30,0
Unterschenkel	5	25,0

### Epidermale Veränderungen unter Dithranol Behandlung

**ABBILDUNG 1:**
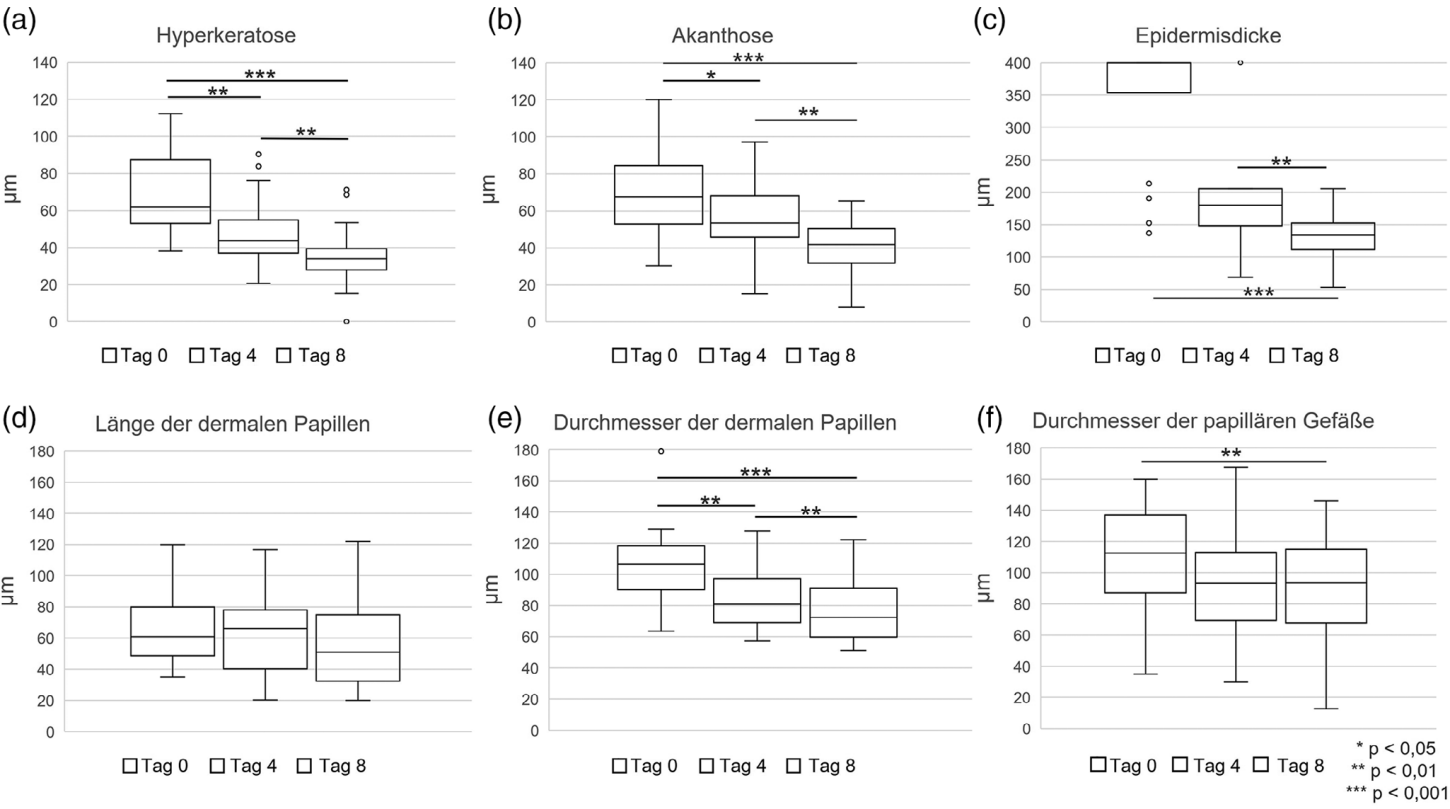
Boxplots zeigen die Ausprägung von (a) Hyperkeratose, (b) Akanthose, (c) Epidermisdicke, (d) Länge der dermalen Papillen, (e) Durchmesser der dermalen Papillen und (f) Durchmesser der papillären Gefäße zu Behandlungsbeginn sowie an Tag 4 und Tag 8 der Dithranoltherapie. Die Messung der Epidermisdicke (c) war auf 400 µm limitiert. Die oberen und unteren Begrenzungen der Boxplots markieren das 25. bzw. 75. Perzentil, der Median ist durch die dicke Linie dargestellt. *p < 0,05; **p < 0,01; ***p < 0,001.

Die KLM‐Merkmale Hyperkeratose, Akanthose und Epidermisdicke reduzierten sich signifikant und deutlich vom Studienbeginn bis zum Tag 8 der Dithranoltherapie. Die zeitlichen Veränderungen der Messwerte (in µm) sind in Abbildung 1a–c dargestellt.

Entsprechend unseren Daten reduzierte sich die mediane Hyperkeratose signifikant um 29,1% (p  =  0,008) von Studienbeginn bis Tag 4 und um 45,0% (p < 0,001) bis Tag 8 (Abbildung 1a). Ebenso zeigte sich eine Reduktion der Akanthose um 21,1% (p  =  0,014) bis Tag 4 und um 38,2% (p < 0,001) bis Tag 8 (Abbildung 1b). Die mediane Epidermisdicke reduzierte sich signifikant um 66,5% (p < 0,001) von Studienbeginn bis Tag 8 (Abbildung 1c). Die Messung der maximalen Epidermisdicke war auf 400 µm begrenzt, ein Wert, der bei 15 Patienten zu Studienbeginn und bei 4 Patienten an Tag 4 erreicht wurde. Daher ist davon auszugehen, dass die tatsächliche Reduktion der Epidermisdicke unter Dithranoltherapie eher unterschätzt wurde.

**ABBILDUNG 2:**
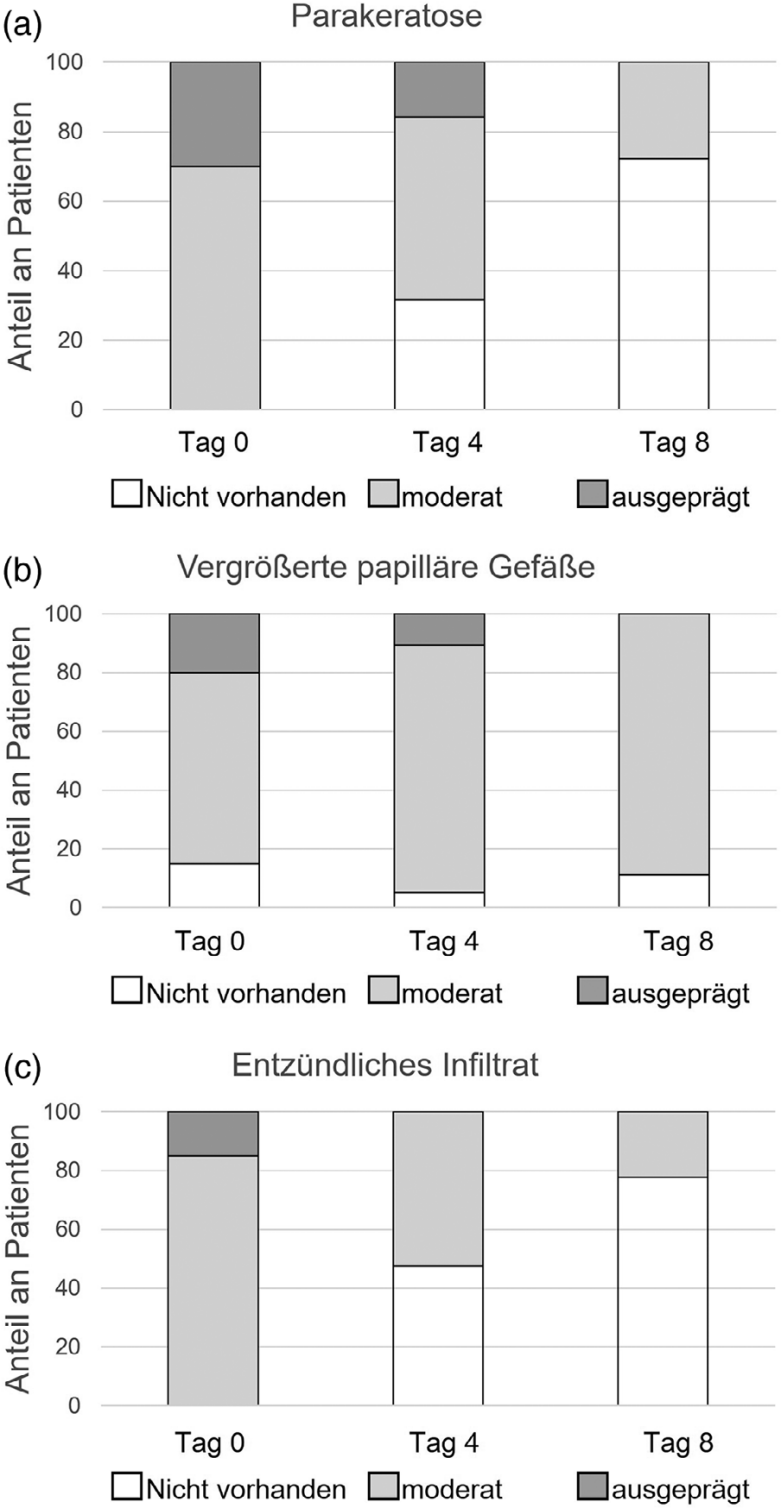
Die Grafik zeigt Vorhandensein von (a) Parakeratose, (b) vergrößerten papillären Gefäßen und (c) Entzündungsinfiltrat in jeweils moderater oder deutlicher Ausprägung beziehungsweise ohne Nachweis zu Behandlungsbeginn sowie an Tag 4 und Tag 8 der Dithranol‐Therapie.

**ABBILDUNG 3:**
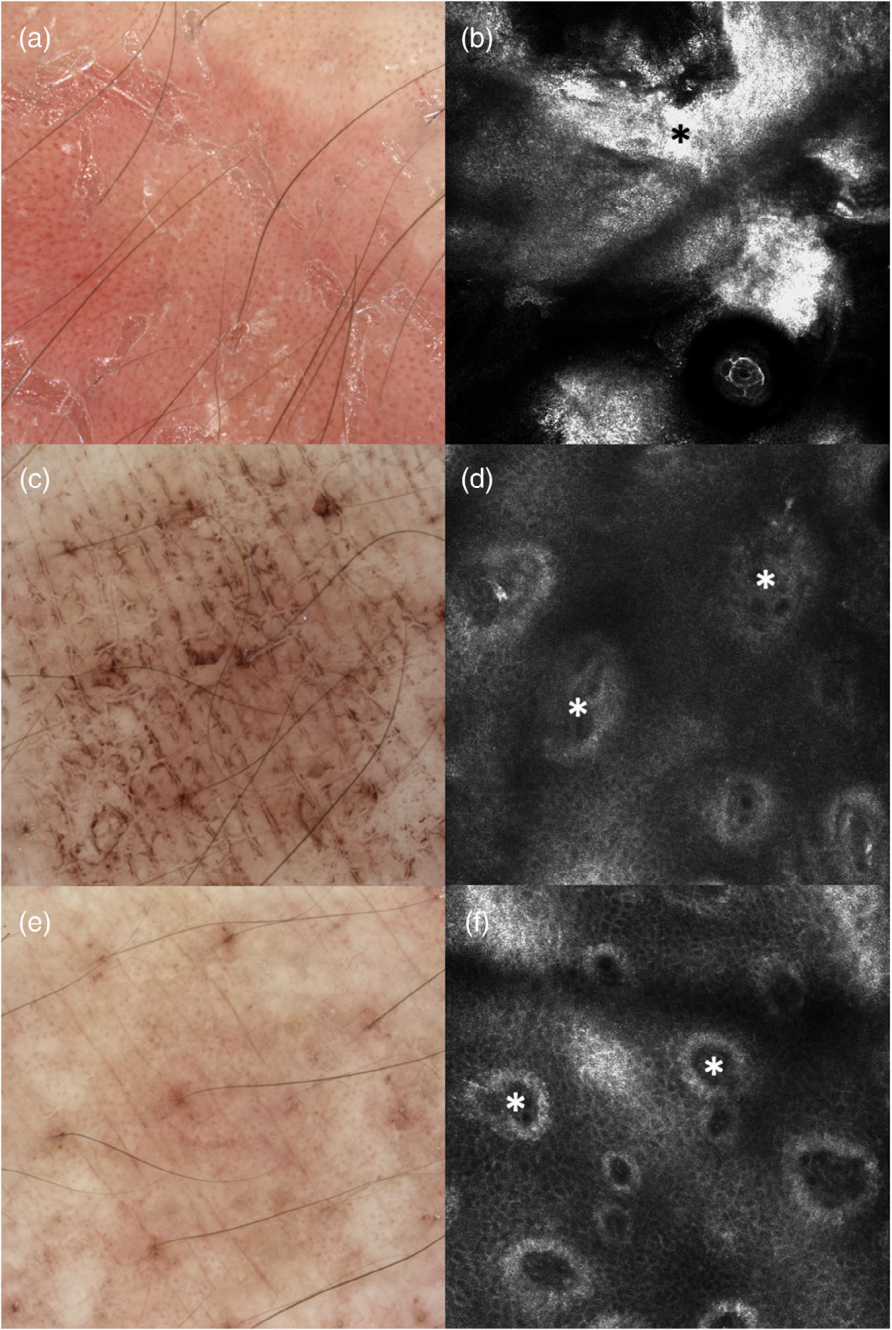
Dermatoskopische Aufnahmen und KLM einer Psoriasisläsion am Unterarm zu (a, b) Behandlungsbeginn, (c, d) an Tag 4 und (e, f) an Tag 8. (a) Die Dermatoskopie zu Behandlungsbeginn zeigt ein Erythem mit regelmäßig verteilten Punktgefäßen und weißer Hyperkeratose, die an (c) Tag 4 und (e) Tag 8 durch eine braune Verfärbung infolge der Dithranol‐Anwendung ersetzt ist. (b) Die KLM zeigt zu Behandlungsbeginn eine Hyperkeratose (schwarzer Stern), an (d) Tag 4 dermale Papillen (weiße Sterne) mit Entzündungszellen und an (f) Tag 8 dermale Papillen mit geringerem Durchmesser (weiße Sterne).

Zu Studienbeginn wurde bei sechs Patienten eine ausgeprägte Parakeratose festgestellt, bei vierzehn Patienten eine moderate Ausprägung. An Tag 8 der Behandlung zeigte kein Patient mehr eine ausgeprägte Parakeratose; bei fünf Patienten wurde eine moderate Ausprägung und bei dreizehn Patienten keine Parakeratose mehr beobachtet (Abbildung 2a). Insgesamt zeigte die semiquantitative Analyse der Parakeratose eine signifikante Reduktion von Studienbeginn bis Tag 8 (p < 0,001). Abbildung 3a, b zeigt eine repräsentative Läsion mit ausgeprägter Hyperkeratose in der KLM. Munro‐Mikroabszesse in moderater Ausprägung wurden lediglich bei einem Patienten zu Studienbeginn und bei einem weiteren Patienten an Tag 4 der Dithranoltherapie festgestellt.

### Dermale Veränderungen unter Dithranoltherapie

**ABBILDUNG 4:**
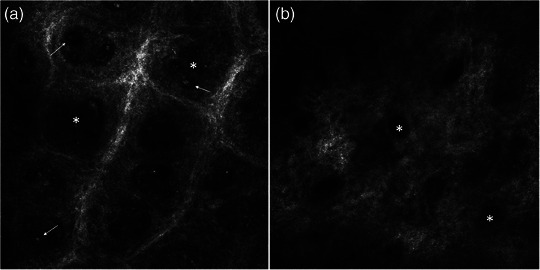
Darstellung einer Psoriasisläsion am Unterarm mittels KLM mit entzündlichem Infiltrat (weiße Pfeile) und dermalen Papillen (weiße Sterne) (a) zu Behandlungsbeginn sowie (b) nach Dithranol‐Therapie. Eine Abnahme sowohl des Infiltrats als auch des Durchmessers der dermalen Papillen ist erkennbar.

Es konnten keine signifikanten Veränderungen in der Länge der dermalen Papillen festgestellt werden (Abbildung 1d). Der mediane Durchmesser der dermalen Papillen verringerte sich um 24,3% (p  =  0,008) von Studienbeginn bis Tag 4 und um 32,1% (p < 0,001) bis Tag 8 (Abbildung 1e). Abbildung 4 zeigt einen repräsentativen Fall mit einer deutlichen Reduktion des Durchmessers der dermalen Papillen unter Dithranoltherapie. Entsprechend reduzierte sich der mediane Durchmesser der papillären Gefäße signifikant um 16,9% (p  =  0,002) von Studienbeginn bis Tag 8 (Abbildung 1f). Zu Studienbeginn waren vergrößerte papilläre Gefäße in deutlicher Ausprägung bei vier Patienten zu finden, in moderater Ausprägung bei 13 Patienten und keine vergrößerten papillären Gefäße bei 3 Patienten. An Tag 8 zeigte keiner der Patienten vergrößerte papilläre Gefäße in deutlichem Ausmaß, 16 in moderatem Ausmaß und zwei Patienten keine vergrößerten papillären Gefäße. Hier waren die Veränderungen von Baseline bis Tag 8 nicht signifikant (p  =  1,0) (Abbildung 2b).

Zu Studienbeginn wurde bei drei Patienten ein ausgeprägtes entzündliches Infiltrat festgestellt, bei siebzehn Patienten ein moderates Infiltrat. An Tag 8 zeigte sich bei keinem Patienten mehr ein ausgeprägtes Infiltrat, bei vier Patienten ein moderates und bei vierzehn Patienten kein entzündliches Infiltrat (Abbildung 2c). Das Ausmaß des entzündlichen Infiltrats nahm signifikant ab – sowohl von Studienbeginn bis Tag 4 (p  =  0,023) als auch bis Tag 8 (p < 0,001). Eine Reduktion der Entzündungszellen im Zentrum der dermalen Papillen, dargestellt mittels KLM, ist in Abbildung 3c–f und Abbildung 4 repräsentativ dargestellt.

### Korrelation mit der Histopathologie

**ABBILDUNG 5:**
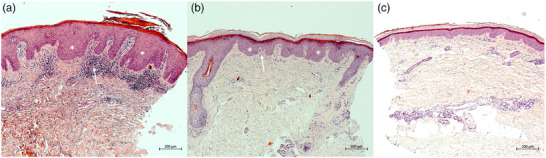
(a) Histologische Darstellung einer Psoriasisläsion zu Behandlungsbeginn, (b) an Tag 4 und (c) an Tag 8 der Dithranoltherapie mit deutlicher Reduktion von Hyperparakeratose, Akanthose (weiße Sterne), Epidermisdicke, erweiterten Kapillaren in den dermalen Papillen sowie Entzündungsinfiltrat (weiße Pfeile) (Hämatoxylin‐Eosin‐Färbung, Originalvergrößerung ×  100).

Die exemplarische Korrelation der KLM‐Aufnahmen mit histopathologischen Schnitten (HE‐Färbungen) von Zielläsionen bei zwei Patienten zeigte zu allen Untersuchungszeitpunkten eine hohe Übereinstimmung. Die Biopsien wurden jeweils unmittelbar nach Durchführung der KLM‐Messungen an identischen Läsionen zu Studienbeginn sowie an Tag 4 und Tag 8 entnommen. Die morphologische Korrelation mit der Histopathologie bestätigte eine Abnahme der Akanthose von Studienbeginn bis Tag 4 und Tag 8 (Abbildung 5). Zusätzlich zeigte die Histopathologie eine graduelle Reduktion der Hyperparakeratose und des entzündlichen lymphozytären Infiltrats (Abbildung 5). Aufgrund des exemplarischen Charakters dieser Korrelation bei nur zwei Patienten erfolgte ausschließlich eine qualitative Analyse; quantitative Auswertungen wurden nicht durchgeführt.

## DISKUSSION

Ziel dieser Pilotstudie war die Erfassung der Effekte einer wiederholten Applikation von Dithranol auf histopathologische Merkmale der Psoriasis vulgaris mittels konfokaler Laserscanmikroskopie, durch die Verfolgung der Veränderungen in einzelnen Psoriasisläsionen. Eine Strategie zur Anwendung der KLM bei Psoriasis wurde bereits zuvor vorgeschlagen.^8^ Frühere Studien haben bereits exzellente Übereinstimmungen zwischen histopathologischen und KLM‐basierten Merkmalen bei Psoriasis gezeigt.^9^, ^10^, ^11^, ^12^ Eine Korrelation zwischen KLM und histopathologischen Befunden bei Psoriasis wurde bereits im Rahmen einer UVB‐Phototherapie belegt.^13^ In dieser Studie wurde die KLM als sehr gut geeignet für das Therapiemonitoring bei Psoriasis bewertet. Darüber hinaus wurde das Therapieansprechen unter systemischer Behandlung (Methotrexat, Acitretin) sowie unter topischer Therapie (Aceclofenac und Betamethason) mittels KLM untersucht.^14^, ^15^

Der biochemische Hintergrund der Wirkung von Dithranol liegt in der Produktion reaktiver Sauerstoffspezies einschließlich Singulett‐Sauerstoff, Superoxid‐Anion‐Radikalen und Hydroxylradikalen.^7^ Dithranol beeinflusst zwei wesentliche Faktoren, die letztlich zum Zelltod führen, wie die Caspase‐Aktivierung und Externalisierung von Phosphatidylserin.^1^ Zusätzlich akkumuliert Dithranol in den Mitochondrien von Keratinozyten, gefolgt von einer Störung des mitochondrialen Membranpotenzials, der Freisetzung von Cytochrom c und der Aktivierung von Caspase‐3.^16^ Therapeutische Effekte von Dithranol wurden bereits in früheren in‐vitro‐Studien gezeigt.^1^, ^7^, ^16^ Unsere Pilotstudie zielte hingegen darauf ab, diese Effekte in vivo mithilfe der KLM zu erfassen. Wir führten eine Pilotstudie durch, da es sich um die erste Untersuchung der Therapieeffekte von Dithranol auf Psoriasis mittels KLM handelt.

Übereinstimmend mit früheren Studien konnten wir zeigen, dass die KLM histomorphologische Korrelate der Psoriasis – wie Akanthose, Hyperkeratose, Parakeratose, erweiterte papilläre Gefäße und ein entzündliches Infiltrat – darstellen kann.^9^ Nach wiederholter Anwendung von Dithranol zeigte sich in der KLM eine Reduktion der Akanthose. Für Dithranol, eines der erfolgreichsten topischen Therapeutika bei Psoriasis, wurde bereits zuvor gezeigt, dass es Apoptose in Keratinozyten induziert.^1^ Wir nehmen an, dass die durch Dithranol induzierte Apoptose die Zahl epidermaler Keratinozyten reduziert und damit zur Abnahme der Akanthose führt. In den KLM‐Aufnahmen fanden wir zudem eine deutlich reduzierte Hyperkeratose und Epidermisdicke. Dies könnte mit der Normalisierung der Zellteilungsrate unter Dithranoltherapie in Zusammenhang stehen. Die Reduktion der Epidermisdicke wurde bereits zuvor als ein Kennzeichen einer effektiven topischen Therapie der Psoriasis gewertet.^15^ In unserer Pilotstudie entwickelten sich die Therapieeffekte der Behandlung mit Dithranol auf die Epidermisdicke kontinuierlich vom Ausgangswert bis zum Tag 8. Erste Effekte zeigten sich dabei bereits sehr rasch, was mit zuvor beschriebenen in‐vitro‐Ergebnissen übereinstimmt.^1^

Zusätzlich zu den oben beschriebenen epidermalen Veränderungen fanden wir unter Dithranol eine Reduktion der Durchmesser der dermalen Papillen. Auch der Durchmesser der papillären Gefäße war unter Dithranol reduziert. Im Gegensatz dazu wurde in früheren Studien unter anderen antipsoriatischen Therapien wie Acetofenac‐Gel oder Betamethason‐Creme keine signifikante Reduktion dilatierter Gefäße beobachtet.^15^

Wir fanden außerdem, dass sich die Parakeratose unter Dithranoltherapie reduzierte, was auf Effekte von Dithranol auf die epidermale Zelldifferenzierung hinweist. In unserer Pilotstudie war das entzündliche Infiltrat mittels KLM gut identifizierbar^17^ und unter Behandlung mit Dithranol reduziert.

Wir untersuchten auch Munro‐Mikroabszesse mittels KLM unter Dithranol‐Behandlung. Allerdings fanden wir diese lediglich bei einem Patienten zu Studienbeginn und bei einem weiteren an Tag 4. In der Literatur werden Munro‐Mikroabszesse deutlich häufiger bei Psoriasis beschrieben.^18^ Dies unterstreicht, dass weitere Studien unsere Ergebnisse bestätigen oder widerlegen müssen. Derzeit werden neue Formulierungen und Applikationsstrategien für Dithranol mithilfe der KLM evaluiert, unter anderem durch Visualisierung der Penetration durch die Haut.^19^, ^20^, ^21^, ^22^, ^23^, ^24^

Eine Limitation bestand in der relativ kleinen Zahl eingeschlossener Patienten in unserer Pilotstudie, die jedoch ausreichte, um wichtige Einblicke in relevante Veränderungen von KLM‐Merkmalen unter Dithranoltherapie zu gewinnen. Eine weitere, allgemeinere Einschränkung der KLM‐Technik liegt darin, dass Untersuchung und Ergebnisse stark vom Trainings‐ und Erfahrungsniveau des Anwenders abhängen. Die erfahrenen Untersucher, die in unserer Pilotstudie die KLM‐Bilder auswerteten, waren weder gegenüber den Patienten noch den Untersuchungstagen verblindet. Zudem wurden alle analysierten KLM‐Merkmale ausschließlich in Psoriasisläsionen untersucht – ein Vergleich mit gesunder, nicht betroffener Haut erfolgte nicht, und es wurde kein spezifischer klinischer Score zur Korrelation herangezogen.

Zusammenfassend zeigte unsere Pilotstudie eine Reduktion pathologischer epidermaler und dermaler Merkmale, die mit Psoriasis assoziiert sind, unter Therapie mit Dithranol. Die KLM erwies sich dabei als geeignete Technik, um diese Merkmale in Korrelation mit der Histopathologie zu untersuchen. Weitere Studien sind jedoch erforderlich, um unsere Ergebnisse zu bestätigen.

## DANKSAGUNG

Open access Veröffentlichung ermöglicht und organisiert durch Projekt DEAL.

## INTERESSENKONFLIKT

H.A. Haenssle hat Honorare und/oder Reisekostenerstattungen von folgenden Unternehmen erhalten, die an der Entwicklung von Geräten zum Hautkrebsscreening beteiligt sind: Scibase AB, FotoFinder Systems GmbH, Heine Optotechnik GmbH, Magnosco GmbH. J.K. Winkler hat ebenfalls Honorare von FotoFinder Systems GmbH erhalten. Die übrigen Autoren geben an, keine Interessenkonflikte zu haben.
